# Rate-Dependent Cohesive Zone Model for Fracture Simulation of Soda-Lime Glass Plate

**DOI:** 10.3390/ma13030749

**Published:** 2020-02-06

**Authors:** Dong Li, Demin Wei

**Affiliations:** 1 School of Civil Engineering and Transportation, South China University of Technology, Guangzhou 510640, China; dmwei@scut.edu.cn; 2 School of Civil Engineering, Guangzhou College of South China University of Technology, Guangzhou 510800, China

**Keywords:** soda-lime glass, cohesive zone model, rate-dependent, impact loading

## Abstract

In this paper, rate-dependent cohesive zone model was established to numerical simulate the fracture process of soda-lime glass under impact loading. Soda-lime glass is widely used in architecture and automobile industry due to its transparency. To improve the accuracy of fracture simulation of soda-lime glass under impact loading, strain rate effect was taken into consideration and a rate-dependent cohesive zone model was established. Tensile-shear mixed mode fracture was also taken account. The rate-dependent cohesive zone model was implemented in the commercial finite element code ABAQUS/Explicit with the user subroutine VUMAT. The fracture behavior of a monolithic glass plate impacted by a hemispherical impactor was simulated. The simulation results demonstrated that the rate-dependent cohesive zone model is more suitable to describe the impact failure characteristics of a monolithic glass plate, compared to cohesive zone model without consideration of strain rate. Moreover, the effect of the strain rate sensitivity coefficient *C*, the mesh size of glass plate and the impact velocity on the fracture characteristics were studied.

## 1. Introduction

Because of its good transparency, soda-lime glass is often used in window panes and curtain wall systems in modern buildings. However, soda lime glass is threatened by extreme loads such as impact loading and blast loading. Under these extreme conditions, due to brittleness, soda-lime glass may suddenly crack, fragment, or even shatter. For example, statistics shows that windborne debris as a major contributor to glass damage when encountering typhoon weather [1]. The study of soda-lime glass materials under impact loading is vital. For this reason, it is necessary to investigate the cracking mechanism of glass window panes.

In recent years, a large number of experimental [2,3,4,5,6,7,8,9,10,11], analytic [12,13,14,15,16], and numerical [17,18,19,20,21,22,23,24,25,26,27,28,29,30,31,32,33,34,35,36,37,38,39,40,41,42,43,44] studies have been devoted to the fracture of soda lime glass. Nie and Chen [2,3,4,5] conducted SHPB experiments to study the effect of the shear stress, mechanical confinement, and temperature on the strength of glass under dynamic loading. Daryadel et al. [6] investigate the influence of the surface flaw on the strength. Johnson et al. [7] and Zhang et al. [9,10,11] reveals that the compressive strength of soda-lime glass is rate-dependent. Further research shows that not only the compressive strength but also the tensile strength could increase with the increase of strain rate [8,11]. Ji and Dharani [15] developed analytic approaches to simulate the damage probability in laminated glass subjected to low velocity small missile impacts. Dharani et al. [14] proposed a two-parameter Weibull distribution to characterize the cumulative probability of inner glass ply breakage. Zhou et al. [13] proposed an empirical formula to calculate the average fragment size. Overend et al. [12] delivered a general crack growth model based on established statistical failure theory and linear elastic fracture mechanics. Nurhuda et al. [16] put forward a model to estimation of strengths in large annealed glass panels which contain widely spaced flaws. A large amount of numerical method is proposed to simulate the cracking process of soda-lime glass. For example, André et al. [39] and Braun et al. [40] adopt the discrete element method (DEM) to conduct the simulation. However, finite element method (FEM) is the most commonly used method. The discontinuity modeling is the most important part when FEM is adopted to the fracture simulation of the soda-lime glass. Element delete method (EDM) is the most common way to model the discontinuity. For instance, Bois et al. [41] simulate an impact of a sphere into a glass plate using an element delete method. Liu et al. [22] adopted EDM to investigate the energy absorption process of windshield under different impact speeds and angles. Extended finite element method (XFEM) [23,24,25,26] is also applied to the fracture simulation of soda-lime glass.

Cohesive zone model (CZM) has been found widely used in the cracking process simulation [27,28,29,30,31,32,33,34,35,36,37,38,44]. Barenblatt [31] and Dugdale [32] were the first to proposed the CZM. After that, application of CZM in the simulation of cracking of glass is more and more widespread. Repetto et al. [28] simulated the dynamic fracture and fragmentation of glass rod under impact loading with CZM. A mixed-mode formula is proposed to combined the tensile and shear cracking mode [29]. Intrinsic and extrinsic [30,34,35,36,37] cohesive zone models were used to simulate the cracking process of laminated glass.

So far, however, there has been little discussion about the rate-dependent cohesive zone model applied to the failure process of soda-lime glass. However, as the experimental results [8,9,10,11] showed that the strength of soda-lime glass could increase with the increase of strain rate. As a result, to ensure the accuracy, a rate-dependent CZM in fracture simulation of soda-lime glass is important and necessary. 

The aim of this study is to improve the accuracy of fracture simulation of glass by employing a rate-dependent CZM. The overall structure of the study takes the form of five sections, including this introductory chapter. Section 2 begins by laying out the theoretical methodology of the rate-dependent cohesive zone model. In Section 3, simulation results are presented, including a successful validation against experimental results of a drop-weight test with monolithic glass plate. Besides, the effects of the strain rate sensitivity coefficient *C*, mesh size and impact velocity are investigated in Section 4.1, Section 4.2 and Section 4.3, respectively. Finally, the conclusions are drawn in Section 5.

## 2. Rate-Dependent Cohesive Zone Model

### 2.1. Traction–separation Law

**Figure 1:**
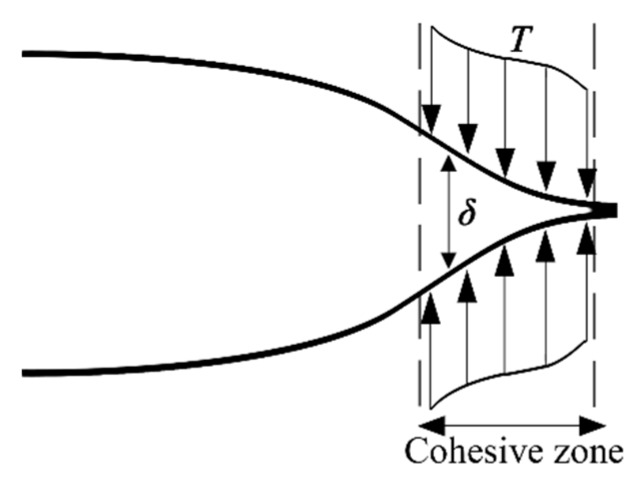
Schematic diagram of cohesive model.

To study the cracking process of soda-lime glass plate under impact loading, the rate-dependent cohesive zone model is employed to simulate the discontinuity in the finite element model. Cohesive zone model assumes that there is a small cohesive zone behind the crack tip, as shown in Figure 1 In the cohesive zone model, there is a mathematical relationship between the separation *δ* and traction *t*, as shown in Equation (1). This mathematical relationship is called traction–separation law (TSL).

*t* = *f*(*δ*) (1)

**Figure 2:**
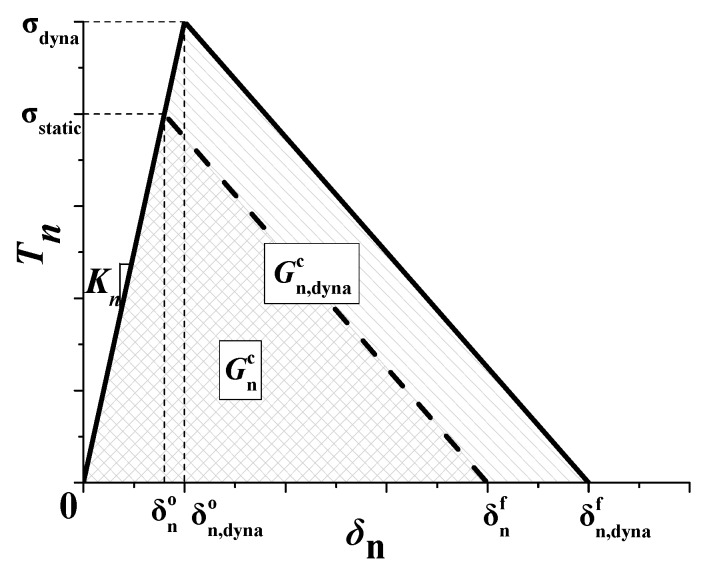
Rate-dependent traction–separation law in normal direction.

A rate-dependent traction–separation law is employed in this research, and its normal law is showed in the Figure 2. The tensile stiffness of the cohesive zone will be degraded when the normal separation *δ*_n_ exceeds to a critical value, $\delta_{n}^{0}$ under quasi-static or $\delta_{n,\mathrm{dyna}}^{0}$ under dynamic for example. As the separation continues to increase to the maximum normal separation ($\delta_{n}^{f}$ under quasi-static and $\delta_{n,\mathrm{dyna}}^{f}$ under dynamic), the tensile stiffness decreases to 0 completely, which indicates the formation of macro cracks. The energy required to form a new crack is called fracture energy. The dissipated energy (*G*) due to failure is equal to the integration of *f*(*δ*_n_) and *δ*_n_. Therefore, the normal fracture energy release rate in Figure 2 is equal to the area enclosed by the traction–separation curve and *δ*_n_ axis. The normal penalty stiffness *K*_n_ is the slope of the curve in the undamaged stage.

**Figure 3:**
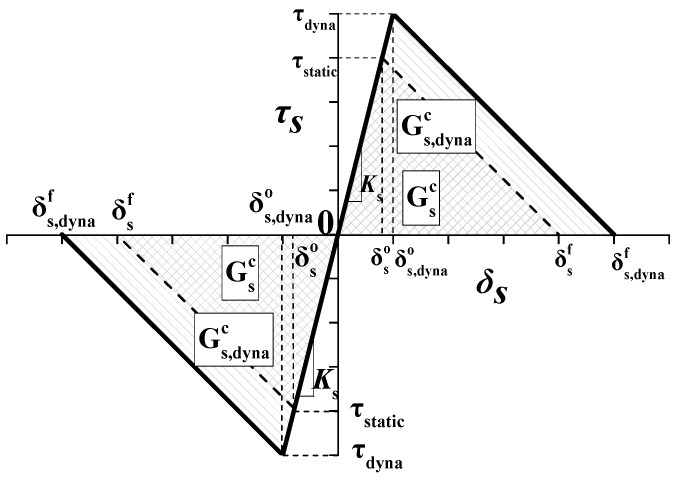
Rate-dependent TSL for shear direction.

Similar to the normal traction–separation law, the shear traction–separation law is defined by a linear relationship between shear traction (*τ*_s_) and the shear separation (*δ*_s_), as shown in Figure 3. The shear stiffness of the cohesive zone will be degraded when the shear separation *δ*_s_ exceeds to a critical value, $\delta_{s}^{0}$ under quasi-static or $\delta_{s,\mathrm{dyna}}^{0}$ under dynamic for example. As the separation continues to increase to the maximum shear separation $\delta_{s}^{f}$ (under quasi-static and $\delta_{s,\mathrm{dyna}}^{f}$ under dynamic), the shear stiffness decreases to 0 completely, which indicates the formation of macro cracks. The dissipated energy (*G*) due to failure is equal to the integration of *f*(*δ*_s_) and *δ*_s_. Therefore, the shear fracture energy release rate in Figure 2 is equal to the area enclosed by the traction–separation curve and *δ*_s_ axis. The shear penalty stiffness *K*_s_ is the slope of the curve in the undamaged stage.

Under dynamic loading, the soda-lime glass is rate dependent [8,10,11]. In JH-2 constitutive model, Holmquist et al. [7] suggested the DIF (dynamic increment factor) should be formulated in Equation (2).

(2) $$DIF=1.0+C\ln(\dot{\varepsilon })$$

where the *C* is the strain rate sensitivity coefficient.

Therefore, the dynamic strength could be expressed by the Equations (3) and (4).

(3) $$\sigma_{\mathrm{dyna}}=DIF\sigma_{\mathrm{static}}$$

(4) $$\tau_{\mathrm{dyna}}=DIF\tau_{\mathrm{static}}$$

The maximum normal separation $\delta_{n}^{f}$ and the maximum shear separation $\delta_{s}^{f}$ under quasi-static could be calculated by the critical energy divided by stress. By multiplying DIF, we could obtain the dynamic maximum separation $\delta_{n,\mathrm{dyna}}^{f}$ and $\delta_{s,\mathrm{dyna}}^{f}$, respectively, as shown in Equations (5) and (6).

(5) $$\delta_{n,\mathrm{dyna}}^{f}=\frac{2G_{n}^{c}}{\sigma_{\mathrm{static}}}DIF$$

(6) $$\delta_{s,\mathrm{dyna}}^{f}=\frac{2G_{s}^{c}}{\tau_{\mathrm{static}}}DIF$$

The dynamic critical energy could be calculated by Equations (7) and (8), according to its definition.

(7) $$G_{n,\mathrm{dyna}}^{c}=2\sigma_{\mathrm{dyna}}\delta_{n,\mathrm{dyna}}^{f}$$

(8) $$G_{s,\mathrm{dyna}}^{c}=2\tau_{\mathrm{dyna}}\delta_{s,\mathrm{dyna}}^{f}$$

### 2.2. Mixed-Mode Failure

The mode mix of the deformation fields in the cohesive zone quantify the relative proportions of normal and shear deformation. Under impact loading, mixed-mode cracking is very common. In mixed-mode failure, the maximum separation until failure $\delta_{m}^{f}$ is expressed by Equation (9).

(9) $$\delta_{m}^{f}=\frac{2G_{m}^{c}}{\sigma_{m}^{0}}$$

where the $G_{m}^{c}$ is mixed-mode critical failure energy, calculated by the Benzeggagh–Kenane fracture criterion [45]. The $G_{m}^{c}$ could be calculated by Equation (10). In Equation (9), the $\sigma_{m}^{0}$ is the mixed-mode separation corresponding the damage initiation, which could be calculated by Equation (11) [29].

(10) $$G_{m}^{c}{=G}_{n,\mathrm{dyna}}^{c}+(G_{s,\mathrm{dyna}}^{c}-G_{n,\mathrm{dyna}}^{c}){\left(\frac{\beta^{2}}{1+\beta^{2}}\right)}^{\eta }$$

(11) $$\sigma_{m}^{0}=K_{n}\delta_{s,\mathrm{dyna}}^{0}\delta_{n,\mathrm{dyna}}^{0}\sqrt{\frac{1+\beta^{2}}{{\left(\delta_{s,\mathrm{dyna}}^{0}\right)}^{2}+{\left(\beta^{2}\delta_{n,\mathrm{dyna}}^{0}\right)}^{2}}}$$

where *β* = $\frac{\delta_{s}}{\delta_{n}^{}}$. $\delta_{n,\mathrm{dyna}}^{0}$ and $\delta_{s,\mathrm{dyna}}^{0}$ are initial damage separations under single failure mode, which could be calculated by Equations (12) and (13).

(12) $$\delta_{n,\mathrm{dyna}}^{0}=\frac{\sigma_{\mathrm{dyna}}}{K_{n}}$$

(13) $$\delta_{s,\mathrm{dyna}}^{0}=\frac{\tau_{\mathrm{dyna}}}{K_{s}}$$

where *K*_n_ and *K*_s_ are the penalty stiffness of normal and shear mode, respectively.

Under mixed-mode failure, the damage is defined as

(14) $$D=\frac{\delta_{m}^{f}\left(\delta_{m}^{\max}-\delta_{m}^{0}\right)}{\delta_{m}^{\max}\left(\delta_{m}^{f}-\delta_{m}^{0}\right)}$$

where the $\delta_{m}^{\max}$ refers to the maximum value of the effective separation $\delta_{m}^{}$ attained during the loading history. $\delta_{m}^{}$ could be expressed by

(15) $$\delta_{m}^{}=\sqrt{{\langle \delta_{n}^{}\rangle }^{2}+{\left(\delta_{s}^{}\right)}^{2}}$$

where $\delta_{n}^{}$ and $\delta_{s}^{}$ are the immediate normal and shear separation, respectively, and <*x*> is the Macauley operator, which could be defined as

(16) $$\langle x\rangle =\{\begin{array}{c} 0{,}_{}^{}x<0\\ x{,}_{}^{}x>0 \end{array}$$

## 3. Simulation Results and Experimental Validation

### 3.1. Set Up of Experimental Test 

**Figure 4:**
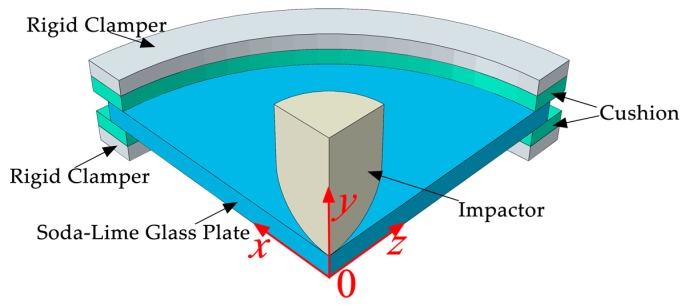
Schematic diagram of basic test device.

Pauw [46] conducted drop-weight test with a monolithic glass specimen, which is a circular soda-lime glass plate with a radius of 50 mm and a thickness of 4 mm. The impactor is a steel cylinder with a 10 mm radius ending in a spherical tip [46]. If the boundary of the fixed plate is too rigid, it is easy to lead to stress concentration, which causes the plate to failure prematurely. Therefore, soft cushions are placed around the upper and lower parts of the plate. The cushions are annular, with a thickness of 3 mm, an inner radius of 46 mm and an outer radius of 54 mm. The schematic diagram of the experimental device is shown in Figure 4. Due to the symmetry, only one-quarter model is showed, and the model is symmetrical about the plane of *x0y* and *y0z.*

During the test, the acceleration time history curve of the punch is recorded by the sensor. The drop height of weight is 200 mm, and the velocity of contact plate is 1.98 m/s. The punch mass is 6.84 kg. The theoretical impact kinetic energy can be calculated as 13407.7 mJ by the equation $E_{k}=\frac{1}{2}mv^{2}$.

### 3.2. Finite Element Model

**Figure 5:**
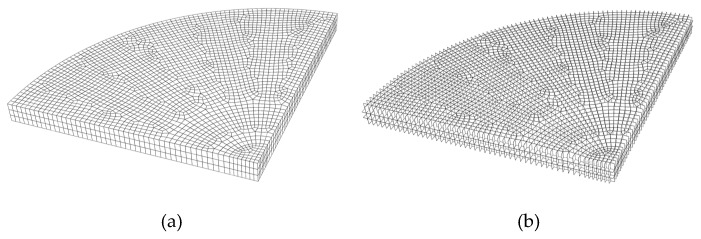
One-quarter mesh model of the plate (**a**) solid element mesh; (**b**) cohesive element mesh.

Eight solid node elements are employed to discrete the soda-lime glass plate. The element size of glass plate is 0.7 mm × 0.7 mm. The mesh model is showed in the Figure 5. Due to the symmetry, only one-quarter model is showed. Because the location of cracks cannot be predicted, zero thickness cohesive elements are inserted between every two solid elements. 

The frictionless hard contact is defined between the impactor and plate, and between the plate and the soft cushion, and between the soft cushion and the rigid clamper. The rigid clamper is fully fitted in translational and rotational degrees of freedom of *x*-, *y*-, and *z*-axis. The boundary conditions of the glass plate and the soft cushion are free. The initial velocity of impactor is 1.98 m/s, which is consistent with the experimental test. Symmetric boundary conditions are set on all symmetric surfaces.

Due to their stiffness is a lot larger than other parts, rigid bodies are employed to model impactor and the clamper. The material of soft cushion is polypropylene and a linear elastic constitutive model is adopted in simulation. Wedge element is used to mesh the impactor. If the size of wedge element is too large, there would be a loss of mass, which could result in the loss of kinetic energy. Table 1 depicts the errors corresponding to different mesh size of impactor. As could be seen in Table 1, the error will become larger when the mesh size of impactor increase. In order to ensure the accuracy, the wedge element size should be controlled lower than 2 mm. In this study, the element size of impactor near the impact area is set to 0.7 mm × 0.7 mm × 0.7 mm, whereas the other area is 2 mm × 2 mm × 2 mm.

### 3.3. Result Analysis

#### 3.3.1. Elastic Result

**Figure 6:**
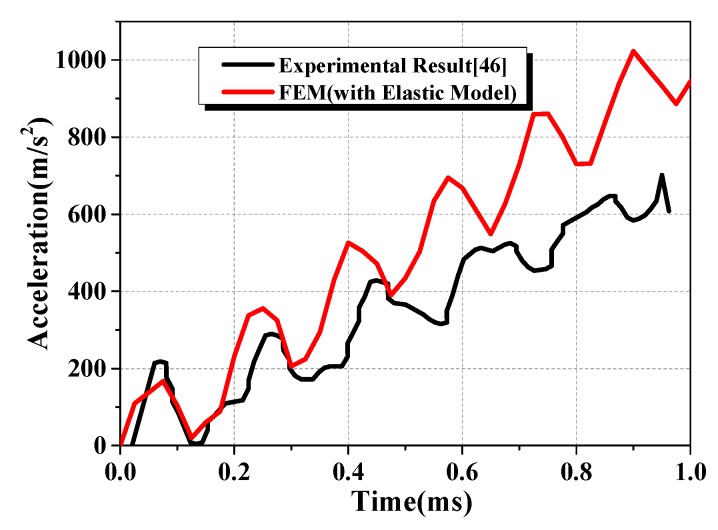
Acceleration-time curves of impactor.

To verify the accuracy of the FE model, including boundary conditions and so on, a simulation with elastic model is conducted. The constants of elastic model are shown in Table 2. Figure 6 shows the acceleration time curves of impactor. Compared to the experimental test results, the simulation result is a bit higher but keep the similar pattern. The reflection of stress wave on the boundary leads to the fluctuation of the curve, which could be found both in the experimental result and the simulation result. 

#### 3.3.2. Rate-Dependent CZM Results

Table 3 depicts the constants of rate-dependent cohesive zone model adopted in this study. In this section, numerical simulations are carried out with the rate-dependent CZM. In the simulation, cohesive element will be removed if damage is equal to 1. The rate-dependent cohesive zone model was implemented in the commercial finite element code ABAQUS/Explicit with the user subroutine VUMAT.

**Figure 7:**
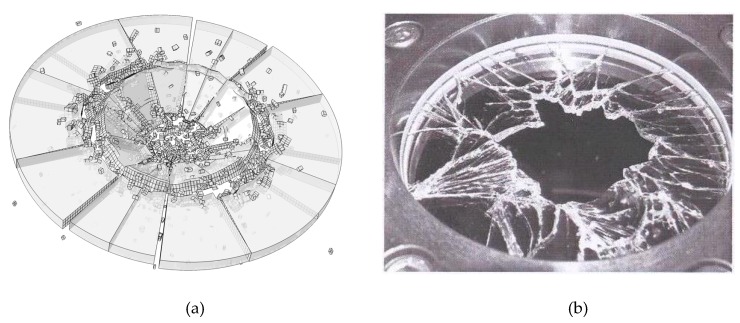
Comparison of the numerical test result and laboratory test result. (**a**) Numerical result; (**b**) experimental result [46].

Figure 7 shows the simulation result conducted with rate-dependent cohesive zone model. The fracture mode of the simulation result agrees well with the experimental result. In the simulation result, the impact area of the plate is found obvious smash. Circle crack is found near the impact area, which thoroughly penetrated the thickness of the plate. Therefore, lots of fragments are formed. In the experimental test results, circle of cracks is also could be found, which results in a large area of vacancy. This phenomenon has also been well simulated in numerical test. In addition, in the experimental test, radial cracks appeared in the plate, which penetrated the whole plate and extended to the edge of the plate. The numerical results have the same crack pattern. To sum up, it can be concluded that the rate-dependent cohesive zone model can accurately simulate the fracture mode of the impact failure of the soda-lime glass plate.

**Figure 8:**
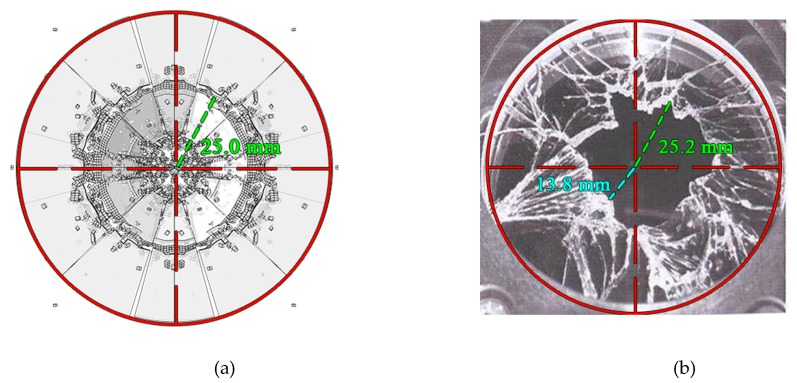
Comparison of the size of fragments. (**a**) Numerical result; (**b**) experimental result [46].

After treated by the image processing method, we could obtain the front view of Figure 7, as shown in Figure 8. The numerical test results are symmetrical. However, due to the irregular microcracks on the surface of the glass plate in the laboratory test, the results of the laboratory test are not symmetrical. In Figure 8a, because of the symmetry, the cracks are regular. The fragments in Figure 8a in the middle are approximately triangular, and the maximum size of the fragment is 25.0 mm. The maximum size of the fragment obtained in the experimental test is about 25.2 mm. Therefore, we could conclude that the rate-dependent cohesive zone model could accurately calculate the size of the fragments under impact loading.

**Figure 9:**
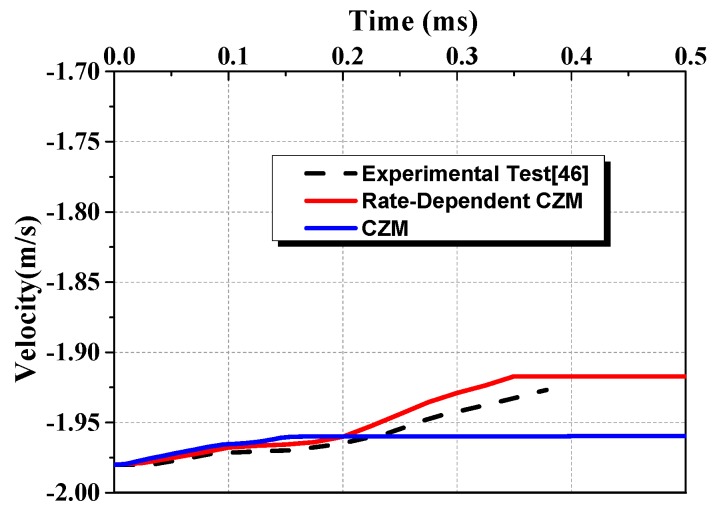
Velocity–time curves of impactor.

Figure 9 depicts the velocity-time curves of impactor. Between 0.0 ms and 0.2 ms, the curve of experimental test, simulation result with rate-dependent CZM and simulation result with CZM agree well. However, after 0.2 ms, the velocity obtained by CZM is obvious lower than the experimental results. The simulation result conducted with rate-dependent CZM is able to predict the velocity well as shown in Figure 9.

**Figure 10:**
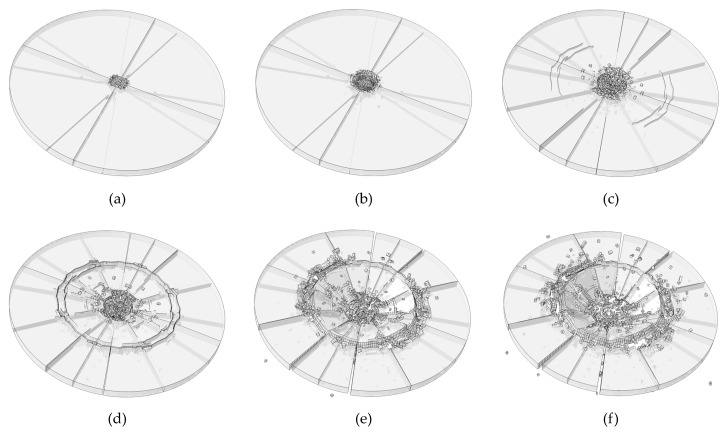
Failure process of the plate (**a**) 0.5 ms; (**b**) 1.0 ms; (**c**) 2.0 ms; (**d**) 2.5 ms; (**e**) 4.0 ms; (**f**) 5.0 ms.

Figure 10 shows the fracture process of the glass plate. As could be seen in the Figure 10a, the radial cracks are the first to appear. After that, inner circle cracks could be found in the impact area, as shown in Figure 10c. Outer circle cracks are found at around 2.0 ms. At this moment, all the cracks are formed basically. We could infer that the formation of radial cracks is mainly because of the discontinuity of angular velocity. The formation of inner circle cracks is because of the punch shear, whereas the formation of outer circle cracks is because of the bending.

## 4. Parametric Studies

### 4.1. Strain Rate Sensitivity Coefficient C

**Figure 11:**
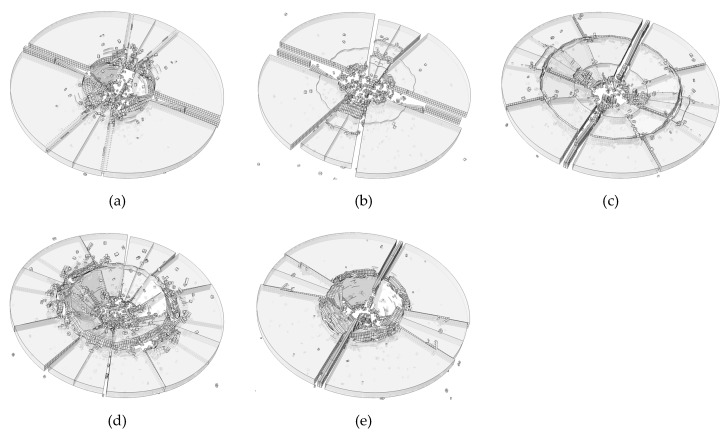
Effect of *C* on the results (**a**) C = 0.00; (**b**) C = 0.01; (**c**) C = 0.02; (**d**) C = 0.03; (**e**) C = 0.04.

In Equation (2), we employed a strain rate sensitivity coefficient *C* to consider the effect of strain rate on the fracture strength. Five numerical tests are carried out in this section, in order to study the effect of *C* on the cracking pattern. The numerical results are shown in Figure 11. In Figure 11a, the coefficient *C* is 0, which indicates that the strength of glass is rate independent. It could be found in Figure 11a that the fracture gathers near the impact area, which is inconsistent with the actual situation shown in Figure 7b. In Figure 11b, most of the energy dissipation is gather near the impact point as well even though the strain rate effect is put into consideration. When the coefficient *C* is 0.02, we could see that the outer circle crack is obvious. The crack pattern when *C* = 0.03 is the most consistent with the experimental result. The strain rate effect forms a shielding zone in the region with high strain rate, which makes the energy propagate outward. However, when *C* = 0.04, the strength of soda-lime glass becomes too high making the fracture area too small compared with Figure 11d. We could also find that the number of small fragments when *C* = 0.04 is obviously less than that in Figure 11d. This can also be attributed to the strength of the glass is too high when *C* = 0.04.

### 4.2. Mesh Size

**Figure 12:**
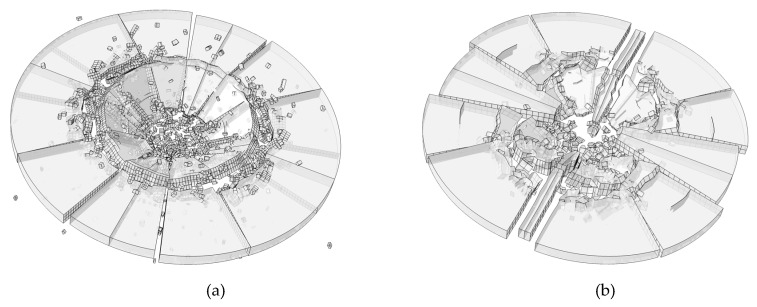
Effect of mesh size on the results. (**a**) Fine mesh; (**b**) coarse mesh.

Two different mesh is conducted in this section, including a fine mesh with element size of 0.7 mm and a coarse mesh with element size of 2 mm. The results are shown in Figure 12. The crack pattern of glass plate is similar. Radial cracks and circle crack can be found in both of numerical result. Fewer small fragments are found in the coarse mesh result. However, the large fragments of both the two results show agreement. We could conclude that the rate-dependent cohesive zone model has little dependence on the mesh size.

### 4.3. Impact Velocity

**Figure 13:**
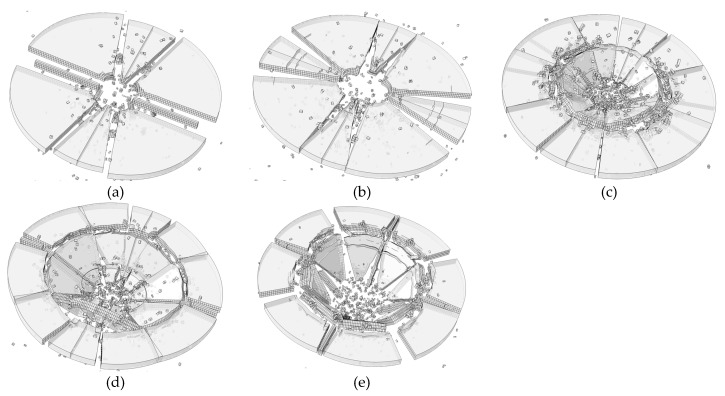
Effect of initial velocity on the results (**a**) v0 = 0.50 m/s; (**b**) v0 = 1.00 m/s; (**c**) v0 = 1.98 m/s; (**d**) v0 = 3.00 m/s; (**e**) v0 = 4.00 m/s.

Figure 13 depicts the numerical results conducted with 5 different impact velocity. When the velocity is 0.50 m/s and 1.00 m/s, no outer circle cracks could be found in the result, as the Figure 13a,b shown. As the velocity increase, the outer circle cracks appear when velocity is 1.98 m/s, 3.00 m/s and 4.00 m/s. Thus, we can conclude that more kinetic energy is needed to form a circular crack compared with a radial crack.

## 5. Conclusions

In this paper, we have established a rate-dependent cohesive zone model for the fracture simulation of soda-lime glass plate. A monolithic glass plate is simulated in this paper conducted with the rate-dependent CZM and the simulation has been validated by the experimental results. Finally, parametric studies are carried out. The main conclusions found of this research are as follows:

(1) Soda-lime glass is a rate dependent material. It is necessary to consider the rate dependence of glass when simulating fracture under impact loading. The numerical simulation results show that the rate-dependent CZM considering the strain rate is more accurate.
(2) The rate-dependent CZM is applied to the finite element simulation by VUMAT. In the rate-dependent CZM, the maximum traction and the maximum separation are rate dependent, which results in that the cohesive element dissipate more energy when it is damaged. The strain rate effect forms a shielding zone in the region with high strain rate, which makes the energy propagate outward.
(3) The rate-dependent cohesive zone model has little dependence on the mesh size. Furthermore, more kinetic energy is needed to form a circular crack compared with a radial crack.

## Figures and Tables

**Table 1:** Relationship between the mesh size of impactor and the kinetic energy

Mesh Size of Impactor (mm)	Kinetic Energy (mJ)	Error (%)
1	13382.3	0.19
2	13277.1	0.97
3	12922.2	3.62
4	12864.9	4.05

**Table 2:** Constants of the linear elastic model for glass and polypropylene

Material	Density (kg/m^3^)	Young’s Modulus (GPa)	Poisson’s Ratio
Soda-lime glass	2530	73	0.3
Polypropylene	1000	2	0.3

**Table 3:** Constants of rate-dependent cohesive zone model.

Properties	Reference Parameters
*σ*_static_	60 MPa [25,37]
*τ*_static_	250 MPa [25,37]
$G_{n}^{c}$	0.01 mJ/mm^2^ [25]
$G_{s}^{c}$	0.05 mJ/mm^2^ [25]
*K*_n_	1.8 × 10^6^ MPa/mm [37]
*K*_s_	6.25 × 10^6^ MPa/mm [37]
*C*	0.03
